# Entropy Maximization, Time Emergence, and Phase Transition

**DOI:** 10.3390/e27060586

**Published:** 2025-05-30

**Authors:** Jonathan Smith

**Affiliations:** Department of Mathematics, Iowa State University, 411 Morrill Rd., Ames, IA 50011, USA; jdhsmith@iastate.edu

**Keywords:** entropy maximization, canonical ensemble, phenomenological rate equation, negative temperature, carrying capacity, order parameter, thermodynamic limit, phase transition, two-person game, predator–prey

## Abstract

We survey developments in the use of entropy maximization for applying the Gibbs Canonical Ensemble to finite situations. Biological insights are invoked along with physical considerations. In the game-theoretic approach to entropy maximization, the interpretation of the two player roles as predator and prey provides a well-justified and symmetric analysis. The main focus is placed on the Lagrange multiplier approach. Using natural physical units with Planck’s constant set to unity, it is recognized that energy has the dimensions of inverse time. Thus, the conjugate Lagrange multiplier, traditionally related to absolute temperature, is now taken with time units and oriented to follow the Arrow of Time. In quantum optics, where energy levels are bounded above and below, artificial singularities involving negative temperatures are eliminated. In a biological model where species compete in an environment with a fixed carrying capacity, use of the Canonical Ensemble solves an instance of Eigen’s phenomenological rate equations. The Lagrange multiplier emerges as a statistical measure of the ecological age. Adding a weak inequality on an order parameter for the entropy maximization, the phase transition from initial unconstrained growth to constrained growth at the carrying capacity is described, without recourse to a thermodynamic limit for the finite system.

## 1. Introduction

Jaynes popularized the entropy maximization technique as a powerful modeling tool for working with finite systems, where results like the Central Limit Theorem or the Stirling Approximation are neither necessary nor appropriate [1,2,3]. On the basis of Jaynes’ work, this survey is designed to highlight some selected aspects of the technique that have appeared over the last thirty years, particularly driven by biological insights in parallel with more traditional topics from physics. Our examples are chosen to be as simple as possible, while still illustrating the key points we wish to convey. In particular, we avoid any reprise of the dependence of entropy and randomness on computational complexity or instrumental resolving power, as discussed in [4]. Further details may be found in the cited references and their bibliographies, but there are many opportunities for interested readers to continue the development and refinement of the topics that we raise.

In Section 2, we set the framework for most of the paper by revisiting the very well-known Gibbs Canonical Ensemble. In particular, we draw attention to an important but rarely mentioned subtlety, namely the strength of the inequality constraints in the procedure of entropy maximization by the method of Lagrange multipliers (7). For perspective, Section 3 takes a brief look at the alternative game-theoretic approach adopted by Topsøe and his school (cf. e.g., [5,6,7]). We propose a biological interpretation for the game as ecological co-evolution between a pair of species: predator and prey. The prey’s interest in randomizing the interactions with the predator, and the predator’s interest in regularizing those interactions, exactly capture the roles of the two players in the abstract game. Thus, the issue,

“The sense in assuming that Player I has the opposite aim, namely to maximize the cost function is more dubious”.

Ref. [5] (p. 198) is resolved by assigning the role of Player I to the prey and Player II to the predator. In this interpretation, the cost function measures how long it will take the predator to determine the prey’s strategy for escape from pursuit.

Section 4 reviews the standard interpretation of the entropy maximization approach to the Canonical Ensemble within statistical mechanics, where macrostates 1,…,i,…,r are identified by respective energies $E_{1},\ldots ,E_{i},\ldots ,E_{r}$. In preparation for the subsequent application to a phase transition in an ecological system (Section 6), we go one step beyond Baez’ advocacy of the Lagrange multiplier β as a “coolness” parameter [8] in preference to the temperature *T*, arguing instead for τ, the negative of the coolness β, as the best choice. Certainly, the temperature *T* is ill-suited to treatment of condensed matter situations where energies of states are bounded both below and above (e.g., in quantum optics, cf. [9,10]). The use of −β as a coordinate in condensed matter physics [9] (Figure 2) then naturally leads to our preference for τ, whose increase is subsequently seen to concur with the Arrow of Time. Compare (27) with (31), for example.

Section 5 uses the Canonical Ensemble for the analysis of an ecology, *Lake Gibbs*, where species 1,…,i,…,r with respective natural growth rates

(1) $$E_{1}<\ldots <E_{i}<\ldots <E_{r}$$

compete within an environment having a fixed carrying capacity of *N* individuals. This system provides a macroscopic model of Eigen’s phenomenological rate equations [11]. While the equations may be solved using standard techniques for handling ordinary differential equations (ODEs), starting from known initial conditions [12,13], the entropy maximization technique offers a novel approach to the solution of the system of coupled ODEs, without the need for initial conditions [14]. This feature of the entropy maximization technique is especially relevant for biological applications, where one encounters existing systems whose genesis is uncertain: the classic “chicken-and-egg” dilemma!

At first glance, use of the Canonical Ensemble in biology may appear to be unrelated to the classical use case of statistical mechanics. However, following the lead of the particle physicists in using natural units with Planck’s constant set to 1 [15] (§III.2), the energies $E_{i}$ that appear in the statistical mechanics applications of the Canonical Ensemble are seen to have the dimensions of inverse time, exactly like the growth rates $E_{i}$ that appear in the ecological application. Thus, our preferred conjugate Lagrange multiplier τ becomes directly identifiable as an emergent time parameter, sharing the statistical macroscopic nature of temperature. As τ increases, the ecology of Lake Gibbs ages by moving from a diverse mix of the species 1,…,r towards an unhealthy monoculture dominated by the most prolific species *r*—compare (1). The ecology could be rejuvenated by restocking the lake with a broad variety of species, thereby resetting the emergent system time τ back to a lower value.

Section 6 extends the entropy maximization treatment of the Lake Gibbs ecology: not only to cover the constrained phase analyzed in Section 5, but also the earlier unconstrained phase where each species *i* (for 1≤i≤r) is growing exponentially at its natural, unchecked pace $E_{i}$, before the carrying capacity of the lake is reached [16]. Thus, entropy maximization is shown to handle a phase transition, for a finite system, without resort to any infinite “thermodynamic limit”. This is achieved by moving beyond the strict inequalities for the constraints on the optimization domain noted in Section 2. Along with positivity constraints for parameters $p_{1},\ldots ,p_{r}$ tracking the respective species 1,…,r, an *order parameter* $p_{0}$ subject to a weak non-negativity constraint is added (38). If the constraint is binding, i.e., $p_{0}=0$, then the entropy maximization reduces to its previous form for the constrained phase as described in Section 5. On the other hand, if the constraint is slack, i.e., $p_{0}>0$, then the entropy maximization returns the unconstrained phase where each species is growing exponentially. As a proof of concept, this basic example suggests that future research, working with richer constellations of strong and weak inequality constraints, should provide finitary entropy maximization analyses of more elaborate, multidimensional phase diagrams.

## 2. The Canonical Ensemble

**Figure 1:**
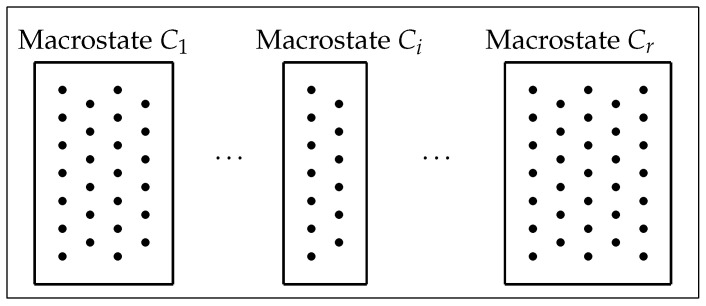
A phase space of microstates, with a partition $\Pi =\left\{\;C_{1},\ldots ,C_{r}\;\right\}$ into macrostates [17] (Figure 2).

Consider a finite, nonempty set or *phase space* (Figure 1) that comprises *N* equally likely individual elements or *microstates* (dots in Figure 1) with a partition

(2) $$\Pi =\left\{\;C_{1},\ldots ,C_{r}\;\right\}$$

into the disjoint union of a family of *r* subsets or *macrostates* (boxes in Figure 1). Suppose that the macrostate $C_{i}$ comprises $n_{i}$ microstates, for 1≤i≤r, so that

(3) $$N=n_{1}+\ldots +n_{r}\;.$$

The set Π of (2) may be considered as (invoking) an *experiment*: take a microstate and determine the macrostate $C_{i}$ to which it belongs.

What information is gained by performing the experiment? Initially, a store of size logN would be required to tag the microstates. Suppose that we perform the experiment and obtain outcome $C_{i}$. In that case, knowing that the microstates are localized within $C_{i}$, we now only require a store of size $\log n_{i}$. The information gain as a result of the experiment is $\log N-\log n_{i}$. However, there is only a probability

(4) $$p_{i}=\frac{n_{i}}{N}$$

of obtaining outcome $C_{i}$. The expected information gain from the experiment is the weighted average

(5) $$H\left(\Pi \right)=\sum_{i=1}^{r}p_{i}\left(\log N-\log n_{i}\right)=-\sum_{i=1}^{r}p_{i}\log p_{i}$$

of the information gains from each of the possible outcomes. This quantity is described as the (information-theoretic) *entropy* of the partition Π. It may be characterized as the expected value of the logarithm of the odds, namely $p_{i}^{-1}$ to one, of obtaining a particular macrostate $C_{i}$.

In practice, the specific partition of the phase space into macrostates is not known a priori. However, suppose that a numerical value (typically representing a dimensioned scientific quantity like an energy or a growth rate) is associated with each macrostate. A particular macrostate $C_{i}$ then consists of all those microstates that yield an observed numerical value $E_{i}$ when the experiment is performed. While the actual probabilities $p_{i}$ of the individual macrostates may be unknown themselves, suppose that the expected outcome

(6) $$E=\sum_{i=1}^{r}p_{i}E_{i}$$

is known, say from a measurement performed on a sample of the microstates. In order to construct a model, the probabilities $p_{i}$ have to be assigned. If the expected value (6) is the only information available, then the truest model is the one that maximizes the entropy H(Π) subject to the constraint (6). This model is known as Gibbs’ *canonical ensemble*.

The maximization problem is usually solved by the method of Lagrange multipliers [18] (Th. 3.2.2). Here, we wish to draw attention to a subtle detail of the procedure, which is rarely stated explicitly: the nonemptiness of the macrostates in the family (2) means that the optimization is taken over the set

(7) $$\Delta_{r-1}^{\circ }=\left\{\;\left(p_{1},\ldots ,p_{r}\right)|p_{1}+\ldots +p_{r}=1,\;0<p_{1},\ldots ,p_{r}\;\right\}$$

of *positive* probabilities, the interior of the (r−1)-dimensional simplex $\Delta_{r-1}$. Thus, in maximization of the Lagrangrian

(8) $$L\left(p_{i},\kappa ,\tau \right)=-\sum_{i=1}^{r}p_{i}\log p_{i}+\sigma \left(1-\sum_{i=1}^{r}p_{i}\right)-\tau \left(E-\sum_{i=1}^{r}p_{i}E_{i}\right)\;,$$

the stationarity conditions

$$\frac{\partial L}{\partial p_{i}}=0$$

for all 1≤i≤r apply since the maximization is performed over $\Delta_{r-1}^{\circ }$. They give

(9) $$\log p_{i}=-\left(1+\sigma \right)+\tau E_{i}$$

or

$$p_{i}=\exp\left(\tau E_{i}\right)/\exp\left(1+\sigma \right)\;.$$

Substitution in the completeness constraint from (3) yields

$$1=\sum_{i=1}^{r}p_{i}=\sum_{i=1}^{r}\exp\left(\tau E_{i}\right)/\exp\left(1+\sigma \right)$$

or

(10) $$\exp\left(1+\sigma \right)=\sum_{i=1}^{r}\exp\left(\tau E_{i}\right)\;.$$

Defining the *partition function* (or “Zustandsumme”)

(11) $$Z\left(\tau \right)=\sum_{i=1}^{r}\exp\left(\tau E_{i}\right)$$

of τ, we have

(12) $$p_{i}=\frac{\exp(\tau E_{i})}{Z(\tau )}$$

in Gibbs’ canonical ensemble. The entropy (5) may be written as

(13) H(Π)=−τE+logZ(τ)

in terms of the variable τ. As another point worthy of special attention, we emphasize that the units for the multiplier τ are inverse to those for the numerical values $E_{i}$.

## 3. The Game Theoretic Approach

While the entropy maximization procedure outlined in the previous section has a number of advantages, most notably the identification of the Lagrange multiplier τ as a conjugate to the numerical values $E_{i}$ assigned to the macrostates, it is not the only approach. The Danish school (cf. [5,6,7], for example) have strongly advocated for an approach that involves a game between two players. In their version,

Player I chooses a consistent distribution, and Player II chooses a general code. …[T]he objective of Player II appears well motivated. [A] cost function can be interpreted as mean representation time, …and it is natural for Player II to attempt to minimize this quantity. The sense in assuming that Player I has the opposite aim, namely to maximize the cost function, is more dubious. [5] (p.198).

Here, in our simplified setting, which is chosen to avoid detailed topological and analytical concerns, we present a brief and introductory alternative account that is founded upon a more adequate, symmetrical notation and a representative interpretation of the two respective Players I and II as prey and predator in an ecological context. A parallel interpretation in a political science context might identify “people” and “government” as the two players. A relationship of this kind is implied, for example, in the work of J.C. Scott [19,20]. We refer to our description as the *Predator–Prey Representation*.

The ecological principle underlying the Predator–Prey Representation is that predators must seek to regularize their relationships with their prey, while the prey seek to randomize those relationships. To catch their prey, predators need encoded hunting strategies. But to evade their predators, the prey need unpredictable escape strategies.

**Figure 2:**
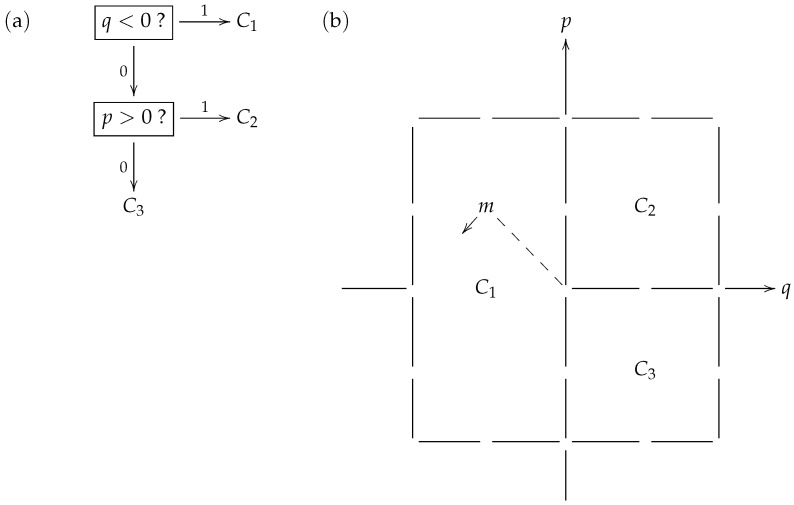
Matching codes to consistent distributions. (**a**): A binary prefix code κ, with 1 as “yes” and 0 as “no” in response to the boxed questions at the internal nodes of the tree. The triple of respective code lengths for $C_{1}=1,C_{2}=01$ and $C_{3}=00$ is (1,2,2). (**b**) A phase space where the partition $\Pi =\left\{\;C_{1},C_{2},C_{3}\;\right\}$ witnesses a consistent distribution, also written as $\Pi =(2^{-1},2^{-2},2^{-2})$ or $2^{-\langle 1,2,2\rangle }$ in an array notation, that matches the code κ. The particular microstate *m* inside the macrostate $C_{1}$ would have the tail of the prey positioned halfway out to the left, with an intermediate value for its leftward momentum.

In the model, the prey may draw from a set $P\subseteq \Delta_{r-1}^{\circ }$ of so-called *consistent* probability distributions Π on a set of evasive actions that includes no more than a finite number *r* of elements. Thus, a consistent distribution gives a specific option for an escape strategy. Figure 2 shows a toy model strategy where the prey is aiming to distract its predator when being pursued from behind, by swinging its tail in a pendulum-like motion. In Figure 2b, the particular consistent distribution Π exhibited is related to (and identified with) a macrostate partition $\Pi =\left\{\;C_{1},C_{2},C_{3}\;\right\}$ of the type of phase space displayed in Figure 1. Furthermore, the phase space in this particular example may be overlaid on the classical phase space of a linear harmonic oscillator (such as a pendulum) with position variable *q* and momentum variable *p*. Thus, a microstate (such as *m* that appears in the macrostate $C_{1}$) may be considered to describe a brief video clip of a specific tail motion.

Table 1 identifies the macrostates with specific tail motion features that would be perceived by the predator in their encoding of the prey’s behavior, using the binary prefix code κ displayed in Figure 2a.

When applying the code κ during the chase in its attempt to learn what the prey is doing, the predator first checks if the prey’s tail is on the left side (q<0). If the answer is “yes” or 1, the predator has taken just 1 step to correctly identify that the prey has selected macrostate $C_{1}$. This event occurs with probability $2^{-1}$ within the prey’s strategy Π. On the other hand, if the answer is “no” or 0, so the tail is on the right, the predator then checks if the prey’s tail is swinging to the right (p>0). If the answer to this question is “yes” or 1, the predator has taken 2 steps to correctly identify that the prey has selected macrostate $C_{2}$, an event that occurs with probability $2^{-2}$ within the strategy Π. Finally, if the answer to the latter question is “no” or 0, the predator has taken 2 steps to correctly identify that the prey has selected macrostate $C_{3}$, an event that, again, occurs with probability $2^{-2}$ within strategy Π. Thus, with the code κ, the expected number of steps that the predator takes to recognize the prey’s strategy Π is

(14) $$\langle \Pi |\kappa \rangle =\frac{1}{2}\cdot 1+\frac{1}{4}\cdot 2+\frac{1}{4}\cdot 2=\frac{3}{2},$$

matching the entropy

$$H\left(\Pi \right)=-\sum_{i=1}^{3}p_{i}\log_{2}p_{i}$$

of the consistent distribution Π in bits.

In the “bra-ket” notation that appears on the left of (14), we switch the sides relative to [5] (p.196) and [7] (8) so that the distribution Π belonging to Player I (the prey) appears first, while the code κ belonging to Player II (the predator) appears second. In general, for a code κ with respective code lengths $\kappa_{1},\ldots ,\kappa_{r}$ and a consistent distribution Π with respective probabilities $p_{1},\ldots ,p_{r}$, the *cost function*

(15) $$\langle \Pi |\kappa \rangle =-\sum_{i=1}^{r}p_{i}\kappa_{i}$$

is defined. As seen on the basis of the illustrative example from Figure 2, the cost function represents the expected time (number of questions asked and answered) taken for Player II using a binary prefix code κ to recognize which macrostate has been chosen from the canonical distribution Π adopted by Player I. The abstract information-theoretic game between Player I and Player II, as envisaged by the Danish school, is instantiated by the concrete ecological games that take place over multiple generations as prey and predator population pairs co-evolve. In particular, the problem raised in the earlier quotation from [5] (p. 198),

“The sense in assuming that Player I has the opposite aim, namely to maximize the cost function is more dubious”,

is clearly solved by the prey’s interest in extending the time it takes its predators to identify an escape strategy.

The full set of escape strategies Π available to the prey is identified as the set P of consistent distributions. Now, consider a particular code κ available to the predator as a hunting strategy. The *risk* [5] (3.11)

(16) $$R\left(P|\kappa \right)=\underset{\Pi \in P}{\sup}\langle \Pi |\kappa \rangle$$

associated with that hunting strategy expresses the maximum length of time it might take the predator to identify the prey’s behavior using κ—a measure of the predator’s risk of starvation if it were to stubbornly rely on κ as its hunting strategy. Successful predators deploy multiple hunting strategies, assembled in a set K of codes κ. Their risk of starvation is reduced to their *minimum risk* value [5] (3.12)

(17) $$R_{\min}\left(P|K\right)=\underset{\kappa \in K}{\inf}R\left(P|\kappa \right)$$

if they are able to draw on any one of these strategies.

Dually, we begin by noting that the full set of hunting strategies κ available to the predator has been identified as the set K of codes. We may now consider a particular consistent distribution Π that is available to the prey as an escape strategy. The *coded entropy*

(18) $$H\left(\Pi |K\right)=\underset{\kappa \in K}{\inf}\langle \Pi |\kappa \rangle$$

(cf. [5] (3.9)) associated with that escape strategy expresses the minimum length of time it might take a predator to identify the strategy—a measure of the prey’s risk of capture or randomization success—if it were to stubbornly rely on Π as its escape strategy. Successful prey species deploy multiple escape strategies, assembled in their repertoire P. Their time of freedom when pursued is maximized to the *maximum coded entropy*

(19) $$H_{\max}\left(P|K\right)=\underset{\Pi \in P}{\sup}H\left(\Pi |K\right)$$

if they are able to draw on any one of these strategies (cf. [5] (3.10)).

Taking infima over K on each side of the quantified statement

$$\forall \;\left(P,\lambda \right)\in P\times K\;,\;\langle P|\lambda \rangle \leq \underset{\Pi \in P}{\sup}\langle \Pi |\lambda \rangle$$

gives $\forall \;P\in P\;,\;\inf_{\kappa \in K}\langle P|\kappa \rangle \leq \inf_{\kappa \in K}\sup_{\Pi \in P}\langle \Pi |\kappa \rangle$. The inequality

(20) $$\underset{\Pi \in P}{\sup}\underset{\kappa \in K}{\inf}\langle \Pi |\kappa \rangle \leq \underset{\kappa \in K}{\inf}\underset{\Pi \in P}{\sup}\langle \Pi |\kappa \rangle$$

then follows on taking the supremum over P. It provides the final link in the full chain

(21) $$\begin{array}{cc} \underset{\kappa \in K}{\inf}\langle \Pi |\kappa \rangle \; & \overset{\left(18\right)}{=}H\left(\Pi |\kappa \right)\leq \underset{\Pi \in P}{\sup}H\left(\Pi |K\right)\overset{\left(19\right)}{=}H_{\max}\left(P|K\right)\\ \; & \overset{\left(20\right)}{\leq }R_{\min}\left(P|K\right)\overset{\left(17\right)}{=}\underset{\kappa \in K}{\inf}R\left(P|\kappa \right)\leq R\left(P|\kappa \right)\overset{\left(16\right)}{=}\underset{\Pi \in P}{\sup}\langle \Pi |\kappa \rangle \end{array}$$

of inequalities that summarizes the relationships between the behaviors of the prey and the predator. Reference [5] continues with a general abstract analysis of when equality is obtained. In the ecological setting, equality is to be expected for stable predator–prey population pairs that have co-evolved over multiple generation times.

## 4. Statistical Mechanics

After the brief excursion into the game-theoretic approach, we return to a consideration of the Lagrangian approach as presented in Section 2. In the classical applications of the canonical ensemble, one may consider the microstates as particles having a certain energy. Thus, the numerical value $E_{i}$ associated with macrostate $C_{i}$ is an energy (say in joules). The conjugate variable τ, which was carefully chosen to match the non-classical applications in the subsequent sections, is connected to the temperature *T* (say in degrees Kelvin) by

(22) (kT)τ+1=0

using Boltzmann’s constant *k*. Baez [8] (p. 30) refers to the traditional conjugate variable β=−τ=1/kT as the *coolness*: the lower the (non-negative) temperature *T*, the higher the value of β. The problem with such traditional conventions, even within statistical mechanics, is that they are ill-adapted to handling *negative temperatures*, which are readily observed in condensed matter situations where energy levels are bounded both below and above [9,10]. In particular, the use of −β (i.e., our τ!) as an abscissa coordinate in the first figure of [9] should be noted. If T=0 and τ=0 are avoided, (22) shows that an increase in τ conveniently corresponds to an increase in *T*, and *vice versa*.

The relation (22) gives some insight into the nature of the quantity τ in the canonical ensemble: Just like the temperature, it is a statistical property of collections of microstates. The *thermodynamic entropy* is

S=kH

in joules per Kelvin degree. The *thermodynamic potential* is the dimensionless quantity

Ψ=logZ(τ),

while the *Helmholtz free energy* is

F=−kTΨ=Ψ/τ

in joules. The relation (13) takes the form

F=E−TS.

The Equation (12) becomes

(23) $$p_{i}=e^{-E_{i}/kT}/e^{-F/kT}\;,$$

a well-known formula of kinetic theory (compare [21]). For example, it may be used to describe the distribution of atmospheric particles at different heights, according to their potential energy in the Earth’s gravitational field [21] (§40-1,2), [3] (§6.1.2(a)).

When considering physical applications of the canonical ensemble, it may prove useful to use *natural units* or *Planck units* with Planck’s constant set to 1 [15] (§III.2). Then, the energies that appear in the statistical mechanics applications of the Gibbs ensemble are seen to have the dimension (time)^−1^. For example, the energy of a photon of light is given as the product of Planck’s constant with the frequency of the corresponding wave.

## 5. Time Emergence

In the statistical mechanical applications of the canonical ensemble discussed in Section 4, the coolness β=1/kT, a Lagrange conjugate of energy, emerges as a statistical property of a collection of microstates. On the other hand, using natural units, such Lagrange conjugates of energy as β or our preferred τ should appear with the units of time. Now, following [14], we examine a model where τ does indeed represent an emergent intrinsic age of a biological system. It provides a conceptually instructive model of competition between *r* different species, labeled 1,…,r, as described by Eigen’s phenomenological rate equations [11]. Suppose that species *i* has an unconstrained growth rate of $E_{i}$ (say in *per annum* units). This means that a population of $n_{i}$ individuals of species *i* growing without constraint has a rate of change

(24) $${\dot{n}}_{i}=n_{i}E_{i}$$

(using Newton’s dot notation for the derivative). At a Newtonian time *t* in an interval [s,u] from a start time *s* to an ultimate time *u*, the population $n_{i}\left(t\right)$ is given as

(25) $$n_{i}\left(t\right)=n_{i}\left(s\right)\exp\left(tE_{i}\right)$$

—exponential growth. Competition (as modeled by Eigen’s equations) arises when the individuals of the *r* species form a joint population maintained at a constant total count *N*. The birth of one individual is compensated for by the death of another.

**Figure 3:**
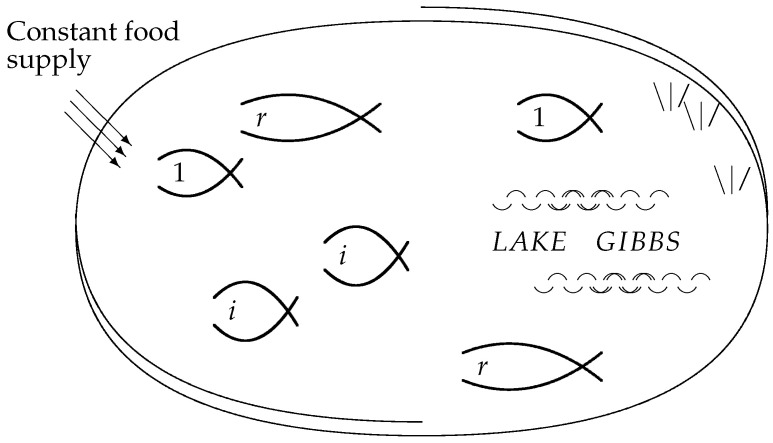
*Lake Gibbs*: a fixed total population *N* of fish in species 1,…,i,…,r competing in an environment with a constant influx of nutrients. Compare [17] (Figure 16), [16] (Figure 1).

Figure 3 visualizes the individuals as fish in a lake, where the food requirements of each individual fish are the same, and the food supply sustains the constant total number *N*. Figure 3 may also be viewed as a slightly less abstract version of Figure 1. The individual fish correspond to the microstates, which share a macrostate if they belong to the same species.

The traditional treatment to determine the population $n_{i}\left(t\right)$ of species *i* at given Newtonian times *t* in an interval [s,u] (cf. [12,13] or [16] (§4)) involves solving the coupled quasilinear system

(26) $${\dot{n}}_{i}\left(t\right)=\left(E_{i}-E\left(t\right)\right)n_{i}\left(t\right)=E_{i}n_{i}\left(t\right)-\sum_{j=1}^{r}n_{i}\left(t\right)\frac{E_{j}}{N}n_{j}\left(t\right)$$

of ordinary differential equations. Although (26) may be seen as a special case of [3] (20 Equation (80)), the treatment in that reference, concerned as it is with equilibrium or long-term values, does not relate to our analysis, the coupling being introduced through the final, quadratic or two-point interaction term of (26). Based on an Ansatz to translate to a linear system [16] (26), the classical solutions

(27) $$n_{i}\left(t\right)=n_{i}\left(s\right)\exp\left(tE_{i}\right)/\exp\left(\int_{s}^{t}E\left(t^{\prime }\right)dt^{\prime }\right)$$

are valid over the Newtonian time interval [s,u]. Here, the initial conditions that are required to solve the system of ordinary differential equations record the population count $n_{i}\left(s\right)$ at t=s.

The function E(t) appearing in the middle part of (26), whose specification follows from

$$0=\frac{d}{dt}\sum_{i=1}^{r}n_{i}\left(t\right)=\sum_{i=1}^{r}{\dot{n}}_{i}\left(t\right)=\sum_{i=1}^{r}\left(E_{i}-E\left(t\right)\right)n_{i}\left(t\right)=\sum_{i=1}^{r}E_{i}n_{i}\left(t\right)-NE\left(t\right)\;,$$

represents the instantaneous *cull rate*

(28) $$E\left(t\right)=\sum_{i=1}^{r}\frac{n_{i}\left(t\right)}{N}E_{i}$$

required to hold the total population constant at the carrying capacity *N*. The argument

$$\int_{s}^{t}E\left(t^{\prime }\right)dt^{\prime }$$

of the exponential in the denominator of the right hand side of (27) is then recognized as counting the total number of starvation victims registered over the time interval [s,t].

In the approach through the canonical ensemble, we imagine going to the lake and catching a moderately sized group of fish in a net. The relative frequency $f_{i}$ of each species *i* in the catch is taken as a good approximation to the overall probability $p_{i}$ of catching a member of that species. In other words, we take

(29) $$f_{i}=\frac{n_{i}}{N}$$

for 1≤i≤r at any given time. Knowing these relative frequencies $f_{i}$, together with the unconstrained growth rates $E_{i}$ of each species, we obtain the expected average value (6) that is the premise for the canonical ensemble. The relationship (28), fundamental to our approach, equates the cull rate *E* to the average

(30) $$\sum_{i=1}^{r}f_{i}E_{i}$$

or *gross growth rate* (GGR) of the population. Equation (12) yields

(31) $$n_{i}\left(\tau \right)=N\exp\left(\tau E_{i}\right)/\sum_{j=1}^{r}\exp\left(\tau E_{j}\right)\;,$$

in particular with

(32) $$n_{i}\left(s\right)=N\exp\left(sE_{i}\right)/\sum_{j=1}^{r}\exp\left(sE_{j}\right)$$

as an initial condition that does not need to be specified separately in our approach. When the species are labeled by increasing unconstrained birth rates (assuming non-degeneracy), say

(33) $$0<E_{1}<E_{2}<\ldots <E_{r}$$

with 1<r, then the most prolific species *r* will ultimately dominate.

The intrinsic time τ that appears in (31) is an emergent statistical property of the complex system. As the system ages, the proportion of the dominant species *r* increases, leading to a lack of biodiversity. Restocking the lake with a good mix of the various species would rejuvenate the ecosystem, resetting the system time τ independently of the relentless forward progress of the Newtonian time *t*.

In conjunction with the emergence of the system time τ, the canonical ensemble treatment of the ecosystem with the fixed carrying capacity *N* has an additional feature, which will be exploited more in the following section. For this discussion, assume the start time *s* is set to t=0, with a uniform distribution at that time in which each species *i* has a population

(34) $$n_{i}\left(0\right)=\frac{N}{r}\;.$$

Then, the solutions (25) of the unconstrained Equation (24) for a negative time *t* take the form

(35) $$n_{i}\left(t\right)=\frac{N}{r}\exp\left(tE_{i}\right)\;.$$

Let *M* denote the total fish population at any given time. Thus, M=N for t>0, while Equation (35) gives

(36) $$M=\frac{N}{r}\sum_{i=1}^{r}\exp\left(tE_{i}\right)$$

for t<0. As a consequence, the equations

(37) $$f_{i}=\exp\left(tE_{i}\right)/\sum_{j=1}^{r}\exp\left(tE_{j}\right)$$

for the relative frequencies, obtained for a positive time *t* from (12) making use of the canonical ensemble description of the constrained ecology, are equally valid during the unconstrained population growth at negative times. It is indeed remarkable that the relative frequencies extrapolate backwards from the canonical ensemble, even though the assumptions leading to the canonical ensemble do not apply in this unconstrained regime.

## 6. A Phase Transition

In the classical entropy-maximization treatment of the canonical ensemble (Section 2), the optimization is taken over the open set

$$\Delta_{r-1}^{\circ }=\left\{\;\left(p_{1},\ldots ,p_{r}\right)|p_{1}+\ldots +p_{r}=1,\;0<p_{1},\ldots ,p_{r}\;\right\}$$

(7) of *positive* probabilities, the interior of the (r−1)-dimensional simplex $\Delta_{r-1}$. In the competition model presented in Section 5, $p_{i}$ represents the probability of catching a fish of species *i*, for 1≤i≤r, although we often prefer to use the relative frequency $f_{i}$ of (29) as a proxy. In this section, following [16], we now consider an additional variable $p_{0}$ and the subset

(38) $$\Delta_{r}^{*}=\left\{\;\left(p_{0},p_{1},\ldots ,p_{r}\right)|p_{0}+p_{1}+\ldots +p_{r}=1,\;0\leq p_{0}\;,\;0<p_{1},\ldots ,p_{r}\;\right\}$$

of the *r*-dimensional simplex $\Delta_{r}$, maximizing the *parametric entropy*

(39) $$-\sum_{i=0}^{r}p_{i}\log p_{i}$$

over the non-open set $\Delta_{r}^{*}$. This procedure leads to a more complete description of the ecology depicted in Figure 3 that also applies to the *unconstrained phase* where the total fish population *M* is below the carrying capacity *N*. In this broadened context, the regime analyzed in Section 5 is called the *constrained phase*. For simplicity of exposition, the phase transition is assumed to take place at system time τ=0 with a uniform distribution of all the species at that time, as in (34) above. Following the analogy between time and temperature, one might regard setting the time of the phase transition to zero as analogous to the (pre-1948) Celsius scale setting of zero for a phase transition of water.

**Figure 4:**
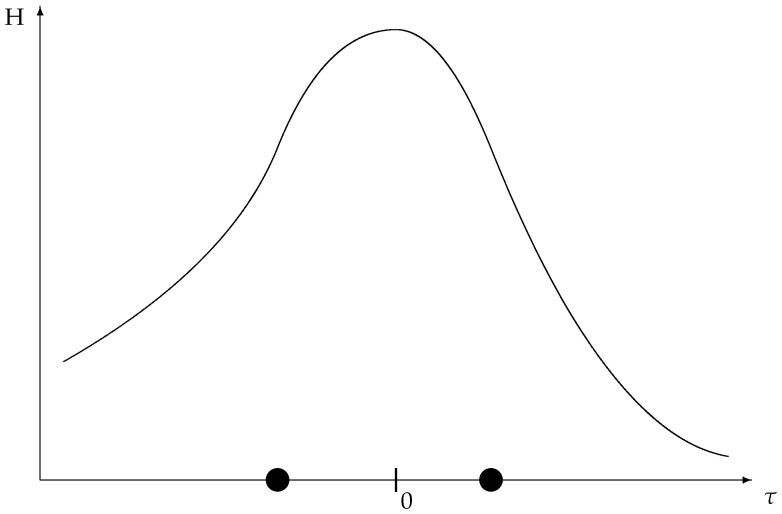
The population entropy (40) over a time range extending from low negative to high positive values, embracing the phase transition at τ=0. At low negative times, the small value of the population entropy is due to the predominance of the least fecund species, required for the population to evolve to the uniform distribution at the phase transition where the carrying capacity is reached. At high positive times, the small value of the population entropy is due to the predominance of the most fecund species. Compare [16] (Figure 3), and also the first figure of [9]. The discs indicate characteristic times for the involution, marking points of inflection for the population entropy. These times do not appear in the first figure of [9], where the entropy curve is concave.

Given the various kinds of phase transition that physicists might recognize, we invoke Penrose’s general definition [22] (§28.1),

“A phenomenon of this nature, where a reduction in the ambient temperature induces an abrupt gross overall change in the nature of the stable equilibrium state of the material, is called a *phase transition*”,

to justify our current terminology. In the ecological setting, a “reduction in the ambient temperature” *T* is interpreted as an increase in the system time τ in accordance with the relation (22). Co-opting common physical terminology, we describe the variable $p_{0}$ in (38) as the *order parameter* [23]. Since the variables $p_{1},\ldots ,p_{r}$ no longer function directly as naive catch probabilities during the unconstrained phase, the way they do during the constrained phase, we refer to them as (additional) *parameters* in the complete history of the ecosystem, thereby leading to the terminology of (39) for the corresponding entropy. We then define

(40) $$H=-\sum_{i=1}^{r}f_{i}\log f_{i}$$

as the *population entropy* (Figure 4) to contrast with the parametric entropy of (39).

Since the population counts of the various fish species do not undergo a discontinuous change at the phase transition, one may wonder where the “abrupt gross overall change in the nature of the stable equilibrium state” comes in. Mathematically, it is seen in the change from the exponential growth solution (25) to the modified version (27), where the denominator with the exponentiated integral suddenly appears. For the individual fish, it means the drastic arrival of the possibility of death by starvation, where previously they were always able to live out their natural lifespans. It is also worth noting the emergence of the “long-range correlations” indicated by the addition of the final term of (26) to the original unconstrained growth Equation (24).

Heuristically, if not too literally, the order parameter $p_{0}$ may be associated with a *ghost species* 0 having a natural unconstrained growth rate $E_{0}=0$. Consider maximization of the parametric entropy (39) over the non-open set $\Delta_{r}^{*}$ of (38) subject to the equality constraint

(41) $$D=\sum_{i=0}^{r}p_{i}E_{i}$$

on the parameters. The quantity *D* appearing in (41)—obviously motivated as a version of (6) that has been extended to include the ghosts—is discussed at the end of this section using (51). We take the Lagrangian

(42) $$L\left(p_{i},\sigma ,\tau \right)=-\sum_{i=0}^{r}p_{i}\log p_{i}+\sigma \left(1-\sum_{i=0}^{r}p_{i}\right)-\tau \left(D-\sum_{i=0}^{r}p_{i}E_{i}\right)$$

in terms of the parameters $p_{0},p_{1},\ldots ,p_{r}$. When the weak inequality constraint on $p_{0}$ from the definition (38) of $\Delta_{r}^{*}$ is *binding* or “active” [18] (p 221), i.e., $p_{0}=0$ and $p_{0}\log p_{0}=0$ (either by convention or as the result of the limiting procedure $\lim_{p_{0}\to 0+}$), we have

$$L\left(p_{0},p_{1},\ldots p_{r},\sigma ,\tau \right)=L\left(p_{1},\ldots p_{r},\sigma ,\tau \right)$$

in terms of the original Lagrangian (8). Thus, in the situation where the order parameter $p_{0}$ is zero and the remaining parameters $p_{i},\ldots ,p_{r}$ are recognized as the corresponding relative frequencies, the extended description reduces to the original description. In particular, when $p_{0}=0$ (i.e., there are no ghosts), the parametric entropy reduces to the population entropy.

When $p_{0}>0$, the weak inequality constraint on $p_{0}$ from the definition (38) of $\Delta_{r}^{*}$ is *slack* or “inactive” [18] (p. 221); the analysis of the Lagrangian (42) for the parametric entropy proceeds in similar fashion to the analysis of the original Lagrangian (8) in Section 2. The stationarity conditions $\partial L/\partial p_{i}=0$ for 0≤i≤r reduce to $\log p_{i}=-\left(1+\sigma \right)+\tau E_{i}$ or $p_{i}=\exp\left(\tau E_{i}\right)/\exp\left(1+\sigma \right)$. A substitution in the completeness constraint yields

$$1=\sum_{i=0}^{r}p_{i}=\sum_{i=0}^{r}\exp\left(\tau E_{i}\right)/\exp\left(1+\sigma \right)$$

or

$$\exp\left(1+\sigma \right)=\sum_{i=0}^{r}\exp\left(\tau E_{i}\right)=1+\sum_{i=1}^{r}\exp\left(\tau E_{i}\right)=1+Z\left(\tau \right)$$

using (11) for the latter term, yielding the expressions

(43) $$p_{i}=\exp\left(\tau E_{i}\right)/\sum_{j=0}^{r}\exp\left(\tau E_{j}\right)=\frac{\exp(\tau E_{i})}{1+Z(\tau )}$$

for the parameters $p_{0},p_{1},\ldots ,p_{r}$.

For i=0, the expression (43) determines the order parameter as

(44) $$p_{0}={\left(1+\sum_{j=1}^{r}\exp\left(\tau E_{j}\right)\right)}^{-1}=\frac{1}{1+Z(\tau )}\;.$$

Taking Equation (43) for 0<i≤r, the remaining parameters may be rewritten in the form

(45) $$p_{i}=p_{0}\exp\left(\tau E_{i}\right)\;.$$

For 1≤i≤r, a substitution of this expression into (35) yields

(46) $$n_{i}=\frac{Np_{i}}{rp_{0}}\;,$$

whence (36) may be rewritten as

(47) $$M=\sum_{j=1}^{r}n_{j}=\frac{N(1-p_{0})}{rp_{0}}$$

to determine the total population in the unconstrained phase in terms of the order parameter. The assignment $p_{0}\mapsto M$ represented by (47) may also be inverted to yield

(48) $$p_{0}=\frac{N}{N+rM}$$

as an equivalent expression of $p_{0}$ in terms of *M*. It is clear that the expressions (46) and (47), valid for negative times, will not continue to hold in the constrained regime where $p_{0}=0$.

In the unconstrained phase, an experiment may be conducted to determine the total population (47) and, thus, the order parameter as given by (48). A fisherman trawls a fixed volume of water and counts the number $M^{\prime }$ of fish caught in the trawl. The number of fish $N^{\prime }$ that would be caught in the trawl at the carrying capacity *N* is presumed to be known, so the total population *M* is obtained as $M^{\prime }N/N^{\prime }$. This refinement of the catch protocol is described as the *trawl*. Using the relation (45) that holds for $p_{0}>0$, the relative frequency of species *i* in the trawl is

(49) $$f_{i}=\frac{\exp(E_{i}\tau )}{\sum_{j=1}^{r}\exp\left(E_{j}\tau \right)}=\frac{p_{i}}{\sum_{j=1}^{r}p_{j}}=p_{i}{\left(1-p_{0}\right)}^{-1}$$

recalling (37) for the first equality. The outer fragment

(50) $$f_{i}=p_{i}{\left(1-p_{0}\right)}^{-1}\;\mathrm{or}\;\mathrm{equivalently}\;p_{i}=f_{i}\left(1-p_{0}\right)$$

of (49) is then seen to hold for the entire history, extending the previous identification $f_{i}=p_{i}$ which only holds in the constrained regime $p_{0}=0$. The factor $\left(1-p_{0}\right)$ appearing in (50) is described as the *modifier* for any $p_{0}$ within the range $\left\{0\right\}\cup \left({\left(1+r\right)}^{-1},1\right)$, and, thus, the parameters $p_{1},\ldots ,p_{r}$ are recognized as *modified relative frequencies*.

The quantity *D* that appears in the constraint (41) may now be examined. We have

(51) $$D=\sum_{i=0}^{r}p_{i}E_{i}=\left(1-p_{0}\right)\sum_{i=1}^{r}f_{i}E_{i}=\left(1-p_{0}\right)E$$

using the second equation of (50). Since *D* is given as the product of the modifier with the gross growth rate (30), it is described as the *modified gross growth rate*. In particular, once the order parameter is known from the trawl, then the modified GGR is obtained from the unmodified GGR, which is also determined from the trawl.

In summary, the extended Lagrangian L given in (42) represents a maximization of the parametric entropy, subject to the completeness constraint on the parameters and the knowledge of the modified gross growth rate *D* that is obtained from the trawl. When L is maximized over the constraint set $\Delta_{r}^{*}$ that includes a weak inequality for the order parameter $p_{0}$ along with the usual strong inequalities for the remaining parameters, it enables one to use entropy maximization for the modeling of a phase transition in a finite situation without recourse to any infinitary “thermodynamic limit”.

## Figures and Tables

**Table 1:** Predator’s interpretation of prey’s tail motion. The prey’s strategy is determined by its random choice of macrostate from the consistent distribution Π shown in Figure 2b.

Macrostate	Positions in the Macrostate	Momenta in the Macrostate
$C_{1}$	Left side	Any
$C_{2}$	Right side	Rightwards
$C_{3}$	Right side	Leftwards

## Data Availability

No new data were created or analyzed in this study.
